# Isolation and Antimicrobial Resistance Patterns of Bacterial Pathogens from Community-Acquired Pneumonia at Adama Hospital Medical College, Adama, Ethiopia

**DOI:** 10.1155/2024/8710163

**Published:** 2024-07-11

**Authors:** Feyissa Hamde, Bayissa Chala, Mesfin Bekele, Abebe Mekuria Shenkutie, Rajiha Abubeker, Ketema Tafess

**Affiliations:** ^1^ Adama Science and Technology University School of Applied Natural Science Department of Applied Biology, Adama, Ethiopia; ^2^ Adama Public Health Research and Referral Laboratory Center, Adama, Ethiopia; ^3^ Department of Health Technology and Informatics The Hong Kong Polytechnic University, Hong Kong, China; ^4^ Ethiopian Public Health Institute, Addis Ababa, Ethiopia; ^5^ Institute of Pharmaceutical Sciences Adama Science and Technology University, Adama, Ethiopia

## Abstract

Community-acquired pneumonia (CAP) is a major cause of morbidity and mortality worldwide. It also contributes significantly to hospital admissions, particularly in low-income countries such as Ethiopia, where it accounts for major public health problems. This could be attributed to the increasing prevalence of antibiotic-resistant pathogens in CAP patients. This study aimed to identify and assess the antibiotic resistance patterns of bacterial isolates from CAP patients at the Adama Hospital Medical College in Adama City, Ethiopia. A cross-sectional study was conducted from November 10, 2022, to November 30, 2023. Demographic, clinical data, and sputum samples were collected from patients with CAP (*n* = 369). Sputum samples were subjected to standard microbiological procedures, including culture, Gram staining, and a panel of different biochemical tests for the identification of pathogenic bacterial isolates. The Kirby–Bauer disc diffusion method was used for drug susceptibility testing. Descriptive statistics were computed by using SPSS (version 26). Of the 369 patients with CAP, bacterial pathogens were identified in 31.7% (*n* = 117, 95% CI: 27.0%–36.7%). The most common isolates were *Moraxella catarrhalis* (*n* = 15; 12.8%), *Staphylococcus aureus* (*n* = 15; 12.8%), *Klebsiella pneumoniae* (*n* = 12; 10.3%), *Escherichia coli* (*n* = 11; 9.4%), *Pseudomonas aeruginosa* (*n* = 11; 9.4%), *Enterobacter species* (*n* = 11; 9.4%), and *Citrobacter species* (*n* = 11; 9.4%). Among the identified isolates, resistance rates were high in Enterobacteriaceae, followed by Gram-positive bacteria, and non-Enterobacteriaceae. Overall, 68 (58.1%) of the identified bacterial isolates were multidrug resistant (MDR), with *K. pneumoniae* accounting for the highest proportion of multidrug resistant isolates (91.7%), while *P. aeruginosa* accounted for the lowest proportion (9.1%) of MDR isolates. This study revealed a high prevalence (31.7%) of bacterial pathogens in CAP patients and higher (58.1%) MDR bacterial pathogens. Therefore, regular surveillance and monitoring systems are warranted for assessing predominant pathogens and antibiotic resistance patterns.

## 1. Introduction

Community-acquired pneumonia is a common cause of morbidity and mortality worldwide, with a significant impact on the healthcare system [1, 2]. The estimated worldwide incidence of CAP varies from 1.5 to 14 cases per 1000 person-years [3, 4]. It is also prevalent in developing countries, causing approximately 700,000 deaths annually [5]. In sub-Saharan Africa, the burden of CAP is increasing, and it is among the major causes of morbidity and mortality among adults [6]. In Ethiopia, although the epidemiology of CAP varies from place to place, a study conducted in the Tigray region revealed a prevalence of 16% in adults [7]. The recent systematic review on the prevalence of CAP in Ethiopia showed that the disease is the second leading cause of death in the country [8].

Community-acquired pneumonia is usually diagnosed by employing a combination of clinical and radiological techniques, and empirical treatment is commonly employed, especially in developing countries [9]. However, it is crucial to understand the specific causes of pneumonia to make informed decisions regarding antibiotic treatment. This is due to variations in underlying causes that can lead to an inadequate response to therapy. Studies conducted in various regions globally have identified bacterial pathogens as common microorganisms responsible for causing CAP [10–12]. The common bacterial pathogens responsible for CAP include *S. pneumoniae*, *H. influenzae*, *E. coli*, *S. aureus*, *M. catarrhalis*, *P. aeruginosa*, and other Gram-negative bacilli [10, 13]. However, the etiology of CAP has undergone a substantial evolution since the preantibiotic era. These etiological variations are due to the irrational use of antibiotics, environmental pollution, advancements in diagnostic techniques and tools, increased awareness of the disease, and widespread introduction of pneumococcal conjugate vaccines [6, 10]. Although *S. pneumoniae* remains the most commonly reported bacterial etiology of CAP worldwide [14], there are reports of an increasing incidence of bacteria such as *M. catarrhalis* [15, 16] and *P. aeruginosa* [17], particularly in immunocompromised patients.

In addition to accurately identifying the causes of CAP, profiling drug resistance of the identified bacterial pathogens plays a crucial role in effectively treating patients. The extensive utilization and misuse of antibiotics have led to a global surge in antibiotic resistance, contributing to the emergence of drug-resistant pathogens [18, 19]. The widespread prevalence of antibiotic resistance and dissemination of MDR strains are major concerns and present significant challenges in the healthcare field [20]. The spread of MDR bacteria within the community has become a critical public health threat, posing considerable difficulties for clinicians owing to high mortality rates and limited treatment options [21]. Studies conducted in some regions of Ethiopia have revealed a high prevalence of mono- and multidrug-resistant bacterial pathogens among patients with CAP [22, 23]. These findings highlight the significance of conducting similar studies in different settings to provide evidence-based interventions for effectively controlling the spread of drug-resistant pathogens.

Therefore, studying the etiological pathogens of CAP and their antibiotic resistance patterns is important to reduce the morbidity and mortality associated with community acquired pneumonia. This study aimed to identify the etiologies of CAP and their antibiotic resistance patterns at Adama Hospital Medical College (AHMC) in Adama City, Ethiopia.

## 2. Materials and Methods

### 2.1. Study Area and Period

The study was conducted at AHMC, Adama, Ethiopia, which is situated in the Oromia region 100 km Southeast of Addis Ababa. AHMC is a referral hospital in Adama and has an estimated catchment of six million individuals from five regions: Oromia, Amhara, Afar, Somali, and Dire-Dawa. With a capacity of 527 beds, the hospital admits and treats an average of 1000 patients daily across 15 outpatient departments, spanning 10 different specialties. The study was conducted from November 10, 2022, to November 30, 2023.

### 2.2. Population and Sample

A total of 369 adults aged ≥18 years who were clinically suspected to have CAP consented to participate in the study. Patients with severe immunosuppression, cough lasting for more than 2 weeks, COVID-19 infection, pregnancy, children under 18 years of age, recent antibiotic use within the past 2 weeks, individuals at risk of hospital-acquired pneumonia, and critically ill patients unable to expectorate sputum were excluded from the study.

### 2.3. Data Collection and Laboratory Methods

Sociodemographic characteristics (age, sex, marital status, family size, educational level, occupation, and monthly income) and pneumonia risk factors such as smoking and travel history, current or past comorbidities, alcohol habits, and prior antibiotic treatment information were collected from patients diagnosed with CAP, presenting with pneumonia-like symptoms or confirmed by X-ray. Additionally, clinical history, physical examination, and laboratory assessment, including initial signs and symptoms of CAP, chest radiograph findings, blood pressure, respiratory rate, pulse rate, oxygen saturation, and vaccination history were recorded. Sputum specimens were obtained from adult patients with CAP by using a sputum cup. Patients were instructed to take deep breaths and then cough deeply and vigorously to produce a minimum of 2 mL of sputum. The sputum cup containing the specimen was appropriately labeled with a unique sample number and immediately transported to the Adama Public Health Research and Referral Laboratory Center for microbiological analysis.

### 2.4. Bacterial Isolation

Isolation and identification of pathogenic bacteria were conducted using conventional microbiological techniques as described in the Clinical Laboratory Standards Institute (CLSI) guidelines [24]. Briefly, a purulent portion of the sputum specimen was inoculated on a Blood Agar Plate (BAP), Chocolate Agar Plate (CAP), and MacConkey Agar Plate (MAP) using a sterile loop. The inoculated CAP and BAP were incubated in a candle jar under 5–10% CO_2_ at 37°C for 24–48 hours. The inoculated MAP was aerobically incubated for 24 h at 37°C. Positive growth on BAP and MAP was subcultured on BAP to identify pure colonies. The pure bacterial isolates were characterized based on colony morphology, hemolysis, Gram staining, and a panel of biochemical tests. Gram-positive bacteria were identified using catalase, coagulase/mannitol salt agar (MSA), optochin, and bile solubility tests, while Gram-negative isolates were identified using the oxidase test, (H_2_S, indole, and motility tests (SIM)), urea test, triple sugar iron agar (TSI), citrate utilization tests, and lysine iron agar (LIA) test. Pure colonies were stored in 20% glycerol at −80°C.

### 2.5. Antibiotic Susceptibility Test

The antibiotic susceptibility test (AST) was performed on positive samples using the Kirby–Bauer disc diffusion method [25]. Three to five morphologically identical pure colonies from an overnight cultured specimen were suspended in 5 ml sterile nutrient broth and thoroughly mixed to obtain a homogeneous suspension. The turbidity of the inoculum was adjusted to 0.5 McFarland standards. Lawn culture, which involves flooding the surface of a solid medium plate with a liquid culture or suspension of bacteria, was performed on Mueller–Hinton agar plates. Antibiotic discs were placed over the lawn culture, and the plates were incubated aerobically or anaerobically at 37°C for 24 h. The strains were tested against various classes of locally used antibiotics, including *β*-lactams (penicillins–10 *μ*g, ampicillin–10 *μ*g, amoxicillin-clavulanate–20/10 *μ*g, and piperacillin tazobactam–100/10 *μ*g), macrolides (azithromycin–15 *μ*g and erythromycin–15 *μ*g), tetracyclines (tetracycline–30 *μ*g), lincomycins (clindamycin–2 *μ*g), glycopeptide (vancomycin–30 *μ*g), sulfonamides (trimethoprim-sulfamethoxazole–25 *μ*g), aminoglycosides (gentamicin–10 *μ*g), amphenicols (chloramphenicol–30 *μ*g), cephalosporins (cefazolin–30 *μ*g, cefuroxime–30 *μ*g, ceftriaxone–30 *μ*g, ceftazidime–30 *μ*g, and cefepime–30 *μ*g), quinolone (ciprofloxacin–5 *μ*g) and carbapenem (imipenem–10 *μ*g and meropenem–10 *μ*g). Finally, the plates were observed for the zone of inhibition and interpreted according to CLSI guidelines, 2021 [24]. Strains were classified as susceptible (S), intermediate (I), or resistant (R). Strains showing resistance to three or more classes of antibiotics were considered to be MDR.

### 2.6. Quality Control

All standard operating procedures (SOPs), including staining reagents, culture media preparation, biochemical tests, and antibiotic susceptibility tests, were strictly followed. The sterility of the prepared culture media was checked by incubating 5% of the batch at 37°C overnight and evaluated for possible bacterial growth. Quality control of all prepared media was also checked by inoculating standard strains such as *E. coli* (ATCC 25922), *S. aureus* (ATCC 25923), *S. pneumoniae* (ATCC 49619), *P. aeruginosa* (ATCC 27853), *P. mirabilis* (ATCC 35659), *K. pneumonia* (ATCCC 700603), *A. baumanni* (ATCC 19606), and *H. influenzae* (ATCC 49766). Furthermore, the potency of the tested antibiotics was evaluated using the control strains. McFarland standard (0.5 McFarland) was used to standardize the inoculum density of the bacterial suspension.

### 2.7. Data Analysis and Interpretation

The data were checked for completeness, coded, and entered into a Microsoft Excel spreadsheet. Subsequently, the data were imported into SPSS version 26 (IBM-SPSS Inc., Chicago, IL, US), coded, and analyzed using descriptive statistics and 95% CI for prevalence.

## 3. Results

### 3.1. Sociodemographic, Clinical, Behavioral Characteristics, and Hospital Outcome of the Study Participants

Of the 369 enrolled patients, 199 (53.9%) were males. The ages of the participants ranged from 18 to 87 years, with a mean (±standard deviation [SD]) of 40.41 [±17.14]. Most of the study participants, 180 (48.8%), were within the age range of 18–35 years. The majority of the participants, 281 (76.2%), were married, and 156 (42.3%) participants were living in a family size of 3–5 members per household. 123 (33.3%) of the participants had no formal education, and more than half 189 (51.2%) of the participants had no monthly income (Table 1). Among these patients, 296 (80.2%) were hospitalized and 73 (19.8%) were treated as outpatients.

A total of 359 (97.3%) patients had cough, 246 (66.7%) reported fatigue, and 60 (16.3%) had a history of prior antibiotic intake in the previous 3 months. No single patient had received the pneumococcal vaccine. Supplementary Table 1 summarizes the most common clinical, behavioral, and comorbidities of patients with CAP.

Vital signs and radiographic findings are presented in Supplementary Table 2. A total of 72 (19.5%) patients were febrile, and 74 (20.0%) experienced hypotension. Among the 283 (76.7%) patients for whom chest X-rays were taken, 133 (36.0%) presented with infiltrate, 83 (22.5%) with consolidation, and 67 (18.2%) with pleural effusion on X-ray.

### 3.2. Types of Bacterial Etiology

**Figure 1:**
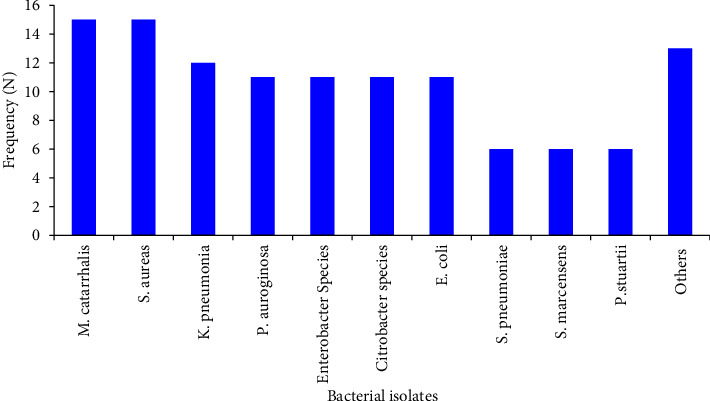
The distribution of bacterial isolates identified from the study participants at AHMC, 2023 (*n* = 369).

The overall bacterial etiology was established in 117 (31.7%; 95% CI: 27.0%–36.7%) patients with CAP. An almost equal frequency of bacterial etiology was identified in males, 59 (50.4%) and females, 58 (49.6%). The predominant isolates were *M. catarrhalis* (*n* = 15; 12.8%) and *S. aureus* (*n* = 15; 12.8%). Other commonly identified bacteria include *K. pneumonia* (*n* = 12; 10.3%); *E. coli* (*n* = 11; 9.4%); *P. aeruginosa* (*n* = 11; 9.4%); *Enterobacter* species (*n* = 11; 9.4%); and *Citrobacter* species, (*n* = 11; 9.4%). Gram-negative bacteria, specifically *Enterobacteriaceae* (*n* = 66, 56.4%) and *non-Enterobacteriaceae* (*n* = 28, 23.9%), were more frequently identified as bacterial pathogens than Gram-positive bacteria (*n* = 23, 19.7%) in patients with CAP (Figure 1).

### 3.3. Antimicrobial Resistance CAP Associates' Isolates

Enterobacteriaceae showed high rates of resistance to cefazolin 59 (89.4%), ampicillin 57 (86.3%), cefuroxime 55 (83.3%), cefepime 54 (81.8%), and ceftriaxone 46 (69.7%). *K. pneumoniae* showed highest levels of resistance to ampicillin 12 (100.0%), cefepime 12 (100.0%), cefazolin 12 (100.0%), ceftriaxone 11 (91.7%), ceftazidime 11 (91.7%), cefuroxime 10 (83.3%), and trimethoprim-sulfamethoxazole 10 (83.3%). *Enterobacter* species were resistant to ampicillin 11 (100.0%) and cefazolin 9 (81.8%). The *Citrobacter* species were resistant to ampicillin 10 (90.9%), cefuroxime 9 (81.8%), cefepime 9 (81.8%), and ceftriaxone 8 (72.7%). *E. coli* demonstrated resistance to cefuroxime 11 (100.0%), cefepime 11 (100.0%), cefazolin 10 (90.9%), and ciprofloxacin 8 (72.7%) (Table 2).

The non-Enterobacteriaceae group exhibited a high resistance to azithromycin 11 (73.3%), erythromycin 9 (60.0%), and cefepime 6 (50.0%). *M. catarrhalis* isolates showed resistance to azithromycin 11 (73.3%), erythromycin 9 (60.0%), and trimethoprim-sulfamethoxazole 8 (53.3%). *P. aeruginosa* isolates demonstrated resistance to cefepime 5 (45.5%) and cefazolin 4 (36.4%) (Table 3).

The Gram-positive bacterial isolates exhibited a high level of penicillin resistance 19 (82.6%). Among the *S. aureus* isolates, all 15 (100%) were resistant to penicillin. In contrast, *S. pneumoniae* isolates were resistant to trimethoprim-sulfamethoxazole 4 (66.7%) and vancomycin 4 (66.7%) (Table 4).

### 3.4. Multidrug Resistance Bacterial Isolates

Multidrug resistance was observed in 68 (58.1%) bacterial isolates. Among the isolated bacteria, multidrug resistance was most prevalent in Enterobacteriaceae 50 (75.8%), followed by Gram-positive bacteria 11 (47.8%) and non-Enterobacteriaceae 7 (21.4%). Among the specific bacterial isolates, the highest rate of multidrug resistance was observed for *K. pneumoniae* 11 (91.7%) (Table 5).

## 4. Discussion

In the present study, bacterial isolates were identified in 31.7% of the CAP patients, which is lower than the findings reported elsewhere in Ethiopia: Dessie town, Northeastern Ethiopia, 46.3% [26]; Gondar, Northwestern Ethiopia, 39.4% [27]; and Jimma town, Southwestern Ethiopia, 50.0% [28]. One possible reason for the lower level of bacterial isolates compared to the previous studies conducted in Ethiopia may be attributed to the widespread usage of antibiotics before presentation to the hospital, as 16.3% of the participants in this study had a history of prior antibiotic intake. On the other hand, the other possible explanation for the lower level of bacterial isolation from CAP patients in our study could also be associated with other causative agents, such as viral causes, which could not be identified by the diagnostic approaches we used in this study. In support of this, study by Jennings et al., [29] and systematic review by Burk et al. [30] showed that viral pathogens could be the etiology of community-acquired pneumonia in 29% and 24.5% of the cases.

Our findings showed a higher prevalence of Gram-negative bacteria compared to Gram-positive bacteria, which is consistent with previous studies conducted in Ethiopia [27, 31, 32]. The changing trend in the etiology of CAP from predominantly Gram-positive bacteria, such as *S. pneumoniae* [14], to Gram-negative bacteria (GNB) could be attributed to several factors. These factors include limited vaccine coverage targeting GNB, inadequate antimicrobial stewardship leading to the irrational use of drugs, and the emergence and spread of drug resistance among GNB [13]. The shift in the trend may also be associated with the production of extended-spectrum *β*-lactamases (ESBL) by Gram-negative bacteria, as the prevalence of ESBL-producing isolates increases, resulting in antimicrobial resistance to drugs [33].

The most frequent isolates in the current study were *M. catarrhalis* and *S. aureus*. This finding is consistent with studies conducted in Uganda, where *Moraxella* species were found to be more prevalent [34] and retrospective observational cohort study conducted in Vienna, Austria [35], where *S. aureus* was the predominant isolate. However, the findings of this study was in variation with the findings conducted in Jimma town, Southwestern Ethiopia [28] where *K. pneumonia* and *P. aeruginosa* were the most frequent isolates followed by other species. The predominant *M. catarrhalis* in this finding showed the significant emergency of this pathogen as an etiology of pneumonia in recent years [36]. The prevalence of *S. aureus* in CAP has also increased in recent decades, with high emergence of new lineages of MRSA in the community [37].

Drug resistance is a significant public health challenge, with the increasing emergence and spread of drug-resistant pathogens. Our study highlights this issue, as a majority of the identified bacterial pathogens demonstrated resistance to currently available drugs. Among the identified bacterial isolates, Enterobacteriaceae exhibited high levels of resistance to ampicillin and cephalosporins, consistent with a study conducted at four different hospitals in Ethiopia [38]. The increasing trend of drug resistance in Enterobacteriaceae is a common observation in Ethiopia [39, 40], likely attributable to the prevalent misuse of drugs without proper prescription by physicians. Our study also showed that *K. pneumoniae* isolates displayed complete resistance to ampicillin, cefepime, and cefazolin, as well as high resistance to ceftriaxone, ceftazidime, cefuroxime, and trimethoprim-sulfamethoxazole. Our findings are in line with the Ethiopian annual antimicrobial surveillance report, which indicated a high resistance of *K. pneumoniae* isolates to cephalosporins and trimethoprim-sulfamethoxazole [41]. *M. catarrhalis* showed 73.3%, 60.0%, and 53.3% resistance rates to azithromycin, erythromycin, and trimethoprim-sulfamethoxazole, whereas *P. aeruginosa* showed 45.5% resistance rate to cefepime. In contrary with our findings, a study done in China showed a high level of sensitivity of *M. catarrhalis* to azithromycin, 100%, and trimethoprim-sulfamethoxazole, 67.7% [11]. The higher resistance pattern observed in this study could be associated with several factors, including the widespread irrational use of drugs prior to patients visiting health facilities and the increasing circulation of drug-resistant isolates in the community warranting continues monitoring of the resistance profile in the community.

Nowadays, bacterial resistance to multiple drugs is becoming a common phenomenon, and our study also supports this fact. In our study, 58.1% of the identified bacterial isolates were found to be MDR. This prevalence is higher compared to a study conducted in Hawassa University Comprehensive Specialized Hospital (HUCSH), Southern Ethiopia [22], which reported an MDR rate of 32.4%. However, the MDR rate in our study is lower than that reported in previous studies conducted in Gondar, Northwestern Ethiopia [27], Felege Hiwot Referral Hospital, Northwestern Ethiopia [23], and Dessie town, Northeastern Ethiopia [26], which reported overall MDR rates of 72.2%, 76.0%, and 84.6%, respectively. One possible explanation for this difference could be geographical variations in bacterial CAP among adults. It is well known that resistance patterns of bacteria can vary geographically owing to factors such as local antimicrobial usage, healthcare practices, and population characteristics. These variations can contribute to the observed differences in the rates of MDR among different bacterial families in different regions or settings.

Our study also revealed that MDR was observed in 75.8% of Enterobacteriaceae isolates, 47.8% of Gram-positive bacteria isolates, and 21.4% of non-Enterobacteriaceae isolates. These findings are comparable to a study conducted at four selected hospitals in central, southern, and northern Ethiopia [38], where Enterobacteriaceae had a higher MDR rate of 83.2%, followed by Gram-positive bacteria (47.8%) and non-Enterobacteriaceae (47.5%). The highest MDR isolates were observed in *K. pneumonia* (91.7%). Similar to our finding, high MDR *K. pneumonia* isolates were also reported from four selected hospitals in central, southern, and northern Ethiopia [38], Tikur Anbessa Specialized Hospital (TASH), Addis Ababa, Ethiopia [41], and Felege Hiwot Referral Hospital, Northwestern Ethiopia [23], with 95.5%, 98.5%, and 100% MDR rates, respectively. Based on our findings, we can speculate that MDR isolates, particularly MDR *K. pneumoniae* isolates, which cannot be treated by commonly used drugs, are widely distributed in various settings in Ethiopia, requiring a concerted effort to control and prevent their spread.

## 5. Conclusions

This study revealed a high prevalence of bacterial pathogens in CAP patients. The antibiotic resistance profile observed in the study area was also found to be alarming. Therefore, it is crucial to strengthen actions aimed at reducing the impact of antimicrobial resistance.

## Figures and Tables

**Table 1:** Sociodemographic characteristics of the study participants, AHMC, 2023 (*n* = 369).

Variable	Category	*N* (%)	Positive *N* (%)	Negative *N* (%)
Gender	M	199 (53.9)	59 (29.6)	140 (70.4)
	F	170 (46.1)	58 (34.1)	112 (65.9)

Age	18–35	180 (48.8)	54 (30.0)	126 (70.0)
	36–49	77 (20.8)	27 (35.1)	50 (64.9)
	50–64	63 (17.1)	17 (27.0)	46 (73.0)
	≥65	49 (13.3)	19 (38.8)	30 (61.2)

Marital status	Single	88 (23.8)	20 (22.7)	68 (77.3)
	Married	281 (76.2)	97 (34.6)	184 (65.4)

Family size	1 to 2	79 (21.4)	25 (31.6)	54 (68.4)
	3 to 5	156 (42.3)	46 (29.5)	110 (70.5)
	6 to 10	115 (31.2)	39 (33.9)	76 (66.1)
	>10	19 (5.1)	7 (36.8)	12 (63.2)

Educational level	Illiterate	123 (33.3)	44 (35.8)	79 (64.2)
	Elementary	158 (42.8)	45 (28.5)	113 (71.5)
	High school	74 (20.1)	25 (33.8)	49 (66.2)
	Coll/Unv.	14 (3.8)	3 (21.4)	11 (78.6)

Occupation	Farmer	104 (28.2)	29 (27.9)	75 (72.1)
	House wife	117 (31.7)	45 (38.5)	72 (61.5)
	G/employee	12 (3.3)	4 (33.3)	8 (66.7)
	Daily laborer	68 (18.4)	19 (27.9)	49 (72.1)
	Student	51 (13.8)	10 (19.6)	41 (80.4)
	Others^*∗*^	17 (4.6)	10 (63.6)	7 (36.4)

Monthly income	<1000 birr per month	189 (51.2)	56 (29.6)	133 (70.4)
	1001–5000 birr per month	94 (25.5)	30 (31.9)	64 (68.1)
	>5000 birr per month	86 (23.3)	31 (36.0)	55 (64.0)

^*∗*^merchant (11, 3%), factory (2, 0.5%), army (1, 0.3%), NGO (1, 0.3%), missionary (1, 0.3%), and commercial sex worker (1, 0.3%).

**Table 2:** Antimicrobial resistance and susceptibility patterns of Enterobacteriaceae isolated from CAP patients, AHMC, 2023 (*n* = 369).

Bacterial isolates	Pattern	Beta lactams/penicillins			Sulfonamides	Aminoglycosides	Amphenicols	Quinolon	1^st^–4^th^ generation cephalosporins					Carbapenem	
									1	2^nd^	3^rd^		4^th^		
		AMP	AMC	PT	TXS	GEN	CHL	CIP	CZL	CRX	CRO	CZ	CFP	IM	MER
*K. pneumonia* (12)	S	—	4 (33.3)	5 (41.7)	1 (8.3)	7 (58.3)	4 (33.3)	1 (8.3)	—	1 (8.3)	—	—	—	4 (33.3)	5 (41.7)
	I	—	3 (25.0)	4 (33.3)	1 (8.3)	—	1 (8.3)	2 (16.7)	—	1 (8.3)	1 (8.3)	1 (8.3)	—	7 (58.3)	3 (25.0)
	R	12 (100.0)	5 (41.7)	3 (25.0)	10 (83.3)	5 (41.7)	7 (58.3)	9 (75.0)	12 (100.0)	10 (83.3)	11 (91.7)	11 (91.7)	12 (100.0)	1 (8.3)	4 (33.3)

*Enterobacter* species (11)	S	—	3 (27.3)	5 (45.5)	3 (27.3)	6 (54.5)	10 (90.9)	1 (9.1)	—	2 (18.2)	4 (36.4)	3 (27.3)	1 (9.1)	3 (27.3)	4 (36.4)
	I	—	5 (45.4)	6 (54.4)	—	1 (9.1)	—	2 (18.2)	2 (18.2)	1 (9.1)	—	3 (27.3)	4 (36.4)	8 (72.7)	4 (36.4)
	R	11 (100.0)	3 (27.3)	—	8 (72.7)	4 (36.4)	1 (9.1)	8 (72.7)	9 (81.8)	8 (72.7)	7 (63.6)	5 (45.4)	6 (54.5)	—	3 (27.3)

*Citrobacter* species (11)	S	—	5 (45.5)	7 (63.6)	5 (45.5)	7 (63.6)	8 (72.7)	1 (9.1)	1 (9.1)	2 (18.2)	2 (18.2)	2 (18.2)	1 (9.1)	6 (54.5)	5 (45.5)
	I	1 (9.1)	2 (18.2)	3 (27.3)	1 (9.1)	3 (27.3)	2 (18.2)	5 (45.5)	1 (9.1)	—	1 (9.1)	4 (36.4)	1 (9.1)	5 (45.5)	4 (36.4)
	R	10 (90.9)	4 (36.4)	1 (9.1)	5 (45.5)	1 (9.1)	1 (9.1)	5 (45.5)	9 (81.8)	9 (81.8)	8 (72.7)	5 (45.5)	9 (81.8)	—	2 (18.2)

*E. coli* (11)	S	3 (27.3)	3 (27.3)	5 (45.4)	4 (36.4)	8 (72.7)	7 (63.6)	1 (9.1)	—	—	1 (9.1)	—	—	6 (54.5)	4 (36.4)
	I	2 (18.2)	5 (45.4)	4 (36.4)	—	—	2 (18.2)	2 (18.2)	1 (9.1)	—	4 (36.4)	4 (36.4)	—	4 (36.4)	7 (63.6)
	R	6 (54.5)	3 (27.3)	2 (18.2)	7 (63.6)	3 (27.3)	2 (18.2)	8 (72.7)	10 (90.9)	11 (100.0)	6 (54.5)	7 (63.6)	11 (100.0)	1 (9.1)	—

*S. marcensens* (6)	S	—	2 (33.3)	3 (50.0)	3 (50.0)	4 (66.7)	5 (83.3)	—	—	1 (16.7)	2 (33.3)	1 (16.7)	—	2 (33.3)	3 (50.0)
	I	—	3 (50.0)	3 (50.0)	—	1 (16.7)	—	2 (33.3)	1 (16.7)	1 (16.7)	—	1 (16.7)	2 (33.3)	4 (66.7)	3 (50.0)
	R	6 (100.0)	1 (16.7)	—	3 (50.0)	1 (16.7)	1 (16.7)	4 (66.7)	5 (83.3)	4 (66.7)	4 (66.7)	4 (66.7)	4 (66.7)	—	—

*P. stuartii* (6)	S	—	2 (33.3)	5 (83.3)	5 (83.3)	5 (83.3)	4 (66.7)	2 (33.3)	—	—	1 (16.7)	1 (16.7)	—	3 (50.0)	2 (33.3)
	I	—	—	1 (16.7)	—	—	1 (16.7)	2 (33.3)	—	1 (16.7)	—	3 (50.0)	1 (16.7)	2 (33.3)	—
	R	6 (100.0)	4 (66.7)	—	1 (16.7)	1 (16.7)	1 (16.7)	2 (33.3)	6 (100.0)	5 (83.3)	5 (83.3)	2 (33.3)	5 (83.3)	1 (16.7)	4 (66.7)

*P. mirabilis* (5)	S	—	1 (20.0)	2 (40.0)	2 (40.0)	5 (100.0)	4 (80.0)	3 (60.0)	—	—	1 (20.0)	—	—	3 (60.0)	3 (60.0)
	I	1 (20.0)	2 (40.0)	2 (40.0)	1 (20.0)	—	—	1 (20.0)	—	—	—	1 (20.0)	1 (20.0)	1 (20.0)	—
	R	4 (80.0)	2 (40.0)	1 (20.0)	2 (40.0)	—	1 (20.0)	1 (20.0)	5 (100.0)	5 (100.0)	4 (80.0)	4 (80.0)	4 (80.0)	1 (20.0)	2 (40.0)

*P. agglomerans* (4)	S	2 (50.0)	1 (25.0)	3 (75.0)	—	2 (50.0)	2 (50.0)	1 (25.0)	—	—	1 (25.0)	—	—	2 (50.0)	2 (50.0)
	I	—	1 (25.0)	—	3 (75.0)	—	1 (25.0)	1 (25.0)	1 (25.0)	1 (25.0)	2 (50.0)	1 (25.0)	1 (25.0)	2 (50.0)	—
	R	2 (50.0)	2 (50.0)	1 (25.0)	1 (25.0)	2 (50.0)	1 (25.0)	2 (50.0)	3 (75.0)	3 (75.0)	1 (25.0)	3 (75.0)	3 (75.0)	—	2 (50.0)

Total (66)	S	5 (7.6)	21 (31.8)	35 (53.0)	23 (34.8)	44 (66.6)	44 (66.6)	10 (15.2)	1 (1.5)	6 (9.1)	12 (18.2)	7 (10.6)	2 (3.0)	29 (43.9)	28 (42.4)
	I	4 (6.1)	21 (31.8)	23 (34.8)	6 (9.1)	5 (7.6)	7 (10.6)	17 (25.8)	6 (9.1)	5 (7.6)	8 (12.1)	18 (27.3)	10 (15.2)	33 (50.0)	21 (31.8)
	R	57 (86.3)	24 (36.4)	8 (12.2)	37 (56.1)	17 (25.8)	15 (22.8)	39 (59.0)	59 (89.4)	55 (83.3)	46 (69.7)	41 (62.1)	54 (81.8)	4 (6.1)	17 (25.8)

Beta lactams (AMP = ampicillin, AMC = amoxicillin-clavulanate, PT = piperacillin tazobactam); sulfonamides (TXS = trimethoprim/sulfamethoxazole); aminoglycosides (GEN = gentamicin); amphenicols (CHL = chloramphenicol); quinolon (CIP = ciprofloxacin); cephalosporins (CZL = cefazolin, CRX = cefuroxime, CRO = ceftriaxone, CZ = ceftazidime, CFP = cefepime); carbapenem (IM = imipenem, MER = meropenem).

**Table 3:** Antimicrobial resistance and susceptibility pattern of non-Enterobacteriaceae bacteria isolated from CAP patients, AHMC, 2023 (*n* = 369).

Bacterial isolates	Pattern	Β-lactams		Sulfonamides	Aminoglycosides	Tetracyclines	Macrolides		Quinolon	Cephalosporins		Carbapenem	
		AMC	PT	TXS	GEN	TE	E	AZ	CIP	CZ	CEP	IM	MER
*M. catarrhalis* (15)	S	14 (93.3)	NA	7 (46.7)	NA	4 (26.7)	6 (40.0)	4 (26.7)	NA	NA	NA	NA	NA
	I	—		—		5 (33.3)	—	—					
	R	1 (6.7)		8 (53.3)		6 (40.0)	9 (60.0)	11 (73.3)					

*P. aeruginosa* (11)	S	NA	8 (72.7)	NA	9 (81.8)	NA	NA	NA	8 (72.7)	7 (63.6)	3 (27.3)	11 (100)	10 (90.9)
	I		3 (27.3)		—				2 (18.2)	—	3 (27.3)	—	1 (9.1)
	R		—		2 (18.2)				1 (9.1)	4 (36.4)	5 (45.5)	—	—

*A. baumannii* (1)	S	NA	1 (100)	1 (100)	1 (100)	NA	NA	NA	—	—	—	1 (100)	—
	I		—	—	—				1 (100)	—	—	—	—
	R		—	—	—				—	1 (100)	1 (100)	—	1 (100)

*B. cepatica* (1)	S	NA	NA	1 (100)	NA	NA	NA	NA	NA	—	NA	NT	1 (100)
	I			—						—			—
	R			—						1 (100)			—

Total (28)	S	14 (87.5)	9 (75.0)	9 (52.9)	10 (83.3)	4 (26.7)	6 (40.0)	4 (26.7)	8 (66.7)	7 (53.8)	3 (25.0)	12 (100)	11 (84.6)
	I	—	3 (25.0)	—	—	5 (33.3)	—	—	3 (25.0)	—	3 (25.0)	—	1 (7.7)
	R	1 (6.7)	—	8 (47.1)	2 (16.7)	6 (40.0)	9 (60.0)	11 (73.3)	1 (8.3)	6 (46.2)	6 (50.0)	—	1 (7.7)

Macrolides (AZ = azithromycin, E = erythromycin), tetracyclines (TE = tetracycline), NA = not applicable, NT = not tested.

**Table 4:** Antimicrobial resistance and susceptibility pattern of Gram-positive bacteria isolated from CAP patients, AHMC, 2023 (*n* = 369).

Bacterial isolates	Pattern	B-lactams	Sulfonamides	Aminoglycosides	Tetracycline	Amphenicols	Macrolides		Lincomycins	Glycopeptide	Quinolon
		PEN	TXS	GEN	TE	CHL	AZ	E	CL	VAN	CIP
*S. aureus* (15)	S	—	11 (73.3)	12 (80)	6 (40.0)	6 (40.0)	4 (26.7)	—	2 (13.3)	NA	5 (33.3)
	I	—	2 (13.3)	1 (6.7)	4 (26.7)	8 (53.3)	5 (33.3)	10 (66.7)	8 (53.3)		5 (33.3)
	R	15 (100)	2 (13.3)	2 (13.3)	5 (33.3)	1 (6.7)	6 (40.0)	5 (33.3)	5 (33.3)		5 (33.3)

*S. pneumonia* (6)	S	3 (50.0)	2 (33.3)	NA	2 (33.3)	1 (16.7)	NA	1 (16.7)	4 (66.7)	—	NA
	I	—	—		2 (33.3)	3 (50.0)		4 (66.7)	1 (16.7)	2 (33.3)	
	R	3 (50.0)	4 (66.7)		2 (33.3)	2 (33.3)		1 (16.7)	1 (16.7)	4 (66.7)	

*C. diphtheria* (2)	S	1 (50.0)	2 (100.0)	2 (100.0)	—	NA	NA	2 (100.0)	2 (100.0)	NA	2 (100.0)
	I	—	—	—	1 (50.0)			—	—		—
	R	1 (50.0)	—	—	1 (50.0)			—	—		—

Total (23)	S	4 (17.4)	15 (65.2)	14 (82.4)	8 (34.8)	7 (33.3)		3 (13.0)	8 (34.8)	—	7 (41.2)
	I	—	2 (8.7)	1 (5.9)	7 (30.4)	11 (52.4)		14 (60.9)	9 (39.1)	2 (33.3)	5 (29.4)
	R	19 (82.6)	6 (26.1)	2 (11.7)	8 (34.8)	3 (14.3)		6 (26.1)	6 (26.1)	4 (66.7)	5 (29.4)

B-lactams (PEN = penicillin); lincomycins (CL = clindamycin); glycopeptide (Van = vancomycin).

**Table 5:** Multidrug-resistant bacterial isolates from CAP patients, AHMC, 2023 (*n* = 369).

Bacterial isolates	Level of resistance (number)								Total MDR isolates ≥R3
	R0	R1	R2	R3	R4	R5	R6	R7	
Enterobacteriaceae (66)		3	13	15	20	7	7	1	50 (75.8)
*K. pneumonia* (12)			1	1	6	1	3		11 (91.7)
*Enterobacter* species (11)			2	1	3	4		1	9 (81.8)
*Citrobacter* species (11)			3	3	4		1		8 (72.7)
*E. coli* (11)		1	3	2	2	1	2		7 (63.6)
*S. marcensens* (6)		1	1	3	1				4 (66.7)
*P. stuartii* (6)			1	3		1	1		5 (83.3)
*P. mirabilis* (5)		1	1	1	2				3 (60.0)
*P. agglomerans* (4)			1	1	2				3 (75.0)
Non-Enterobacteriaceae (28)	8	8	4	6	1				7 (21.4)
*M. catarrhalis* (15)	3	3	4	4	1				5 (33.3)
*P. aeruginosa* (11)	5	5		1					1(9.1)
*A. baumannii* (1)				1					1(100)
*B. cepatica* (1)		1							0 (0.0)
Gram-positive bacteria (23)	1	7	4	4	2	3	2		11 (47.8)
*S. aureas* (15)		5	2	3	1	3	1		8 (53.3)
*S. pneumonia* (6)		2	1	1	1		1		3 (50.0)
*C. diphtheria* (2)	1		1						0 (0.0)
Total (117)	9	18	21	25	24	9	9	1	68 (58.1)

R0: susceptible to all antibiotics, R1–R7: resistance to 1, 2, 3, 4, 5, 6, and 7 antibiotics, ≥R3: resistance to 3 or more antibiotics.

## Data Availability

Data generated in this study are included within the manuscript or supplementary information files.

## Supplementary Materials

Supplementary Materials Supplementary Table 1: clinical and behavioral characteristics of the study participants, AHMC, 2023 (*n* = 369). Supplementary Table 2: vital signs and laboratory findings at the time of admission, at AHMC, 2023 (*n* = 369).
