# Supplementary material for: Isolation and Antimicrobial Resistance Patterns of Bacterial Pathogens from Community-Acquired Pneumonia at Adama Hospital Medical College, Adama, Ethiopia
Source: J Trop Med. 2024 Jul 11;2024:8710163. doi: 10.1155/2024/8710163 (PMC11257760; doi:10.1155/2024/8710163)
Supplement: Supplementary Materials — Supplementary Table 1: clinical and behavioral characteristics of the study participants, AHMC, 2023 (n = 369). Supplementary Table 2: vital signs and laboratory findings at the time of admission, at AHMC, 2023 (n = 369). [file 8710163.f1.docx]

**Isolation and Antimicrobial Resistance Patterns of Bacterial Pathogens from Community Acquired Pneumonia at Adama Hospital Medical College, Adama, Ethiopia**

Feyissa Hamde^1^, Bayissa Chala^1^, Mesfin Bekele^2^, Abebe Mekuria^3^, Rajiha Abubeker^4^, Ketema Tafess ^1&5*^

^1^Adama Science and Technology University, School of Applied Natural Science, Department of Applied Biology, Adama, Ethiopia

^2^Adama Public Health Research and Referral Laboratory Center, Adama, Ethiopia

^3^Department of Health Technology and Informatics, The Hong Kong Polytechnic University, Hong Kong SAR, China

^4^Ethiopian Public Health Institute, Bacterial, Parasitic and Zoonotic Research Directorate, Addis Ababa, Ethiopia

^5^Institute of Pharmaceutical Sciences, Adama Science and Technology University, Adama, Ethiopia

**Supplementary Table 1:** Clinical and behavioral characteristics of the study participants, AHMC, 2023 (n=369).

| **Variable** | **Category** | **Frequency(N)** | **Percentage (%)** |
| --- | --- | --- | --- |
| Symptoms | Cough | 359 | 97.3 |
|  | Fatigue | 246 | 66.7 |
|  | Loss of appetite | 217 | 58.8 |
|  | Breathing difficulty | 97 | 26.3 |
|  | Headache | 70 | 19.0 |
|  | Myalgia | 65 | 17.6 |
|  | Vomiting | 39 | 10.6 |
|  | Irritability | 5 | 1.4 |
|  | Coma | 5 | 1.4 |
|  | Sore throat | 3 | 0.8 |
|  | Convulsion | 2 | 0.5 |
| Smoking habit | Non-smoker | 342 | 92.7 |
|  | Occasional smoker | 13 | 3.5 |
|  | Frequent smoker | 7 | 1.9 |
|  | Ex-smoker | 7 | 1.9 |
| Drink alcohol | Never | 319 | 86.5 |
|  | Occasionally | 37 | 10 |
|  | Regularly | 13 | 3.5 |
| Comorbidities | Asthma | 52 | 14.1 |
|  | Hypertension | 20 | 5.4 |
|  | Respiratory disease | 17 | 4.6 |
|  | Diabetes mellitus | 10 | 2.7 |
|  | Heart disease | 10 | 2.7 |
|  | COPD | 8 | 2.2 |
|  | Measles | 6 | 1.6 |
|  | Kidney problem | 6 | 1.6 |
|  | Cancer | 5 | 1.4 |
|  | CNS disease | 3 | 0.8 |
|  | Epilepsy | 2 | 0.5 |
| Patient history | Infection in last 3 months | 46 | 12.5 |
|  | Hospitalized in last 3 months | 28 | 7.6 |
|  | Travel history | 9 | 2.4 |

**Supplementary Table 2:** Vital signs and laboratory findings at the time of admission, at AHMC, 2023 (n=369).

| **Vital signs Value** | |
| --- | --- |
| Body temperature (⁰C) mean (SD) | 36.6 (±.39) |
| Hypothermia (≤36.0 ⁰C), n | 43 (11.7) |
| Normal (36.0-38.4 ⁰C), n | 254 (68.8) |
| Fever (≥38.5 ⁰C), n | 72 (19.5) |
| Respiratory rate, n (SD) | 20.8 (±3.8) |
| Respiratory rate < 20 n | 249 (67.5) |
| Tachypnea (respiratory rate >20), n | 120 (32.5) |
| Systolic blood pressure, (mmHg), mean (SD) | 111.4 (±17.1) |
| Hypotension (< 100 mmHg), n | 74 (20.0) |
| Normal (100-140 mmHg), n | 277 (75.1) |
| Hypertension (> 140 mmHg), n | 18 (4.9) |
| Diastolic blood pressure, mean % (SD) | 69.5 (±12.8) |
| Hypotension (< 60 mmHg), n | 68 (18.4) |
| Normal (60-89mmHg), n | 269 (72.9) |
| Hypertension (≥90 mmHg), n | 32 (8.7) |
| Heart rate, mean (SD) | 96.4(±17.7) |
| Bradycardia (heart rate < 60 bpm), n | 1 (0.3) |
| Normal (60-99 bpm), n | 220 (59.6) |
| Tachycardia (heart rate ≥ 100 bpm), n | 148 (40.1) |
| Hemoglobin, mean (SD) | 12.4 (±5.4) |
| Severe anemia (Hb <7g/dl), n | 23 (6.2) |
| Moderate anemia (Hb 7-9.9 g/dl), n | 59 (16.0) |
| Mild anemia (Hb 10-11.9 g/dl), n | 71 (19.3) |
| Normal (Hb ≥ 12 g/dl), n | 216 (58.5) |
| Oxygen saturation mean % (SD) | 95.3(±3.6) |
| Hypoxemia (SpO2 < 90%), n | 22 (6.0) |
| Normal (SpO2 90-100), n | 346 (93.7) |
| Hyperoxemia (SpO2 >100%), n | 1 (0.3) |
| X-ray finding |  |
| Consolidate | 83 (22.5) |
| Infiltrate | 133 (36.0) |
| Pleural effusion | 67 (18.2) |
